# Exploring Australian pharmacists' views toward reducing the risk of medicines‐related harm in aged care residents

**DOI:** 10.1002/prp2.1104

**Published:** 2023-05-24

**Authors:** Sheraz Ali, Colin M. Curtain, Gregory M. Peterson, Mohammed S. Salahudeen

**Affiliations:** ^1^ School of Pharmacy and Pharmacology, College of Health and Medicine University of Tasmania Hobart Australia

**Keywords:** aged care resident, harm, medicines‐related harm, medicines‐related injury, older resident

## Abstract

Medicines‐related harm is common in older people living in residential aged care facilities (RACFs). Pharmacists offering services in the aged care sector may play a key role in reducing medicines‐related injury. This study aimed to explore Australian pharmacists' views toward reducing the risk of medicines‐related harm in older residents. Qualitative, semi‐structured interviews were conducted with 15 Pharmacists across Australia providing services (e.g., through the provision of medication reviews, supplying medications, or being an embedded pharmacist) to RACFs identified via convenience sampling. Data were analyzed by thematic analysis using an inductive approach. Medicines‐related harm was thought to occur due to polypharmacy, inappropriate medicines, anticholinergic activity, sedative load, and lack of reconciliation of medicines. Pharmacists reported that strong relationships, education of all stakeholders, and funding for pharmacists were facilitators in reducing medicines‐related harm. Pharmacists stated that renal impairment, frailty, staff non‐engagement, staff burnout, family pressure, and underfunding were barriers to reducing medicines‐related harm. Additionally, the participants suggested pharmacist education, experience, and mentoring improve aged care interactions. Pharmacists believed that the irrational use of medicines increases harm in aged care residents, and medicines‐specific (e.g., sedative load) and patient‐specific risk factors (e.g., renal impairment) are associated with injuries in residents. To reduce medicines‐related harm, the participants highlighted the need for increased funding for pharmacists, improving all stakeholders' awareness about medicines‐associated harms through education, and ensuring collaboration between healthcare professionals caring for older residents.

**Abbreviations**
CNS: central nervous system
COVID‐19: coronavirus disease 2019
GPs: general practitioners
IQR: interquartile range
PIMs: potentially inappropriate medications
RACF: residential aged care facilities
RMMRs: Residential Medication Management Reviews
STOPP/START: Screening Tool of Older People's Prescriptions and Screening Tool to Alert to Right Treatment

## 1 INTRODUCTION

Medicines‐related harm is any injury resulting from the use of medicines.^1^, ^2^ Advancing age is associated with an increased risk of medicines‐related harm, such as falls, fractures, delirium, functional decline, cognitive impairment, hospitalization, and mortality.^3^ The combination of factors such as old age, multiple comorbidities, and polypharmacy, increases the risk of medicines‐related injury in older people.^4^ Older people in residential aged care facilities (RACFs) are usually the frailest, with multiple comorbidities and functional and cognitive impairment.^5^ Moreover, polypharmacy is common in older residents, as half of this cohort usually take nine or more medicines.^6^ In the United States, the occurrence of medicines‐related harm in RACFs is as high as 10.8 events per 100‐resident months.^7^ Medicines‐related harm constitutes a significant imposition on the Australian healthcare system,^8^ where the annual cost of medication‐related hospital admissions is approximately $1.4 billion, which is equivalent to 15% of total Pharmaceutical Benefits Scheme expenditure.^9^, ^10^ In 2022, it was estimated that 250 000 Australians are hospitalized every year due to medicines‐related harm.^9^ Around one in five hospitalizations among older Australians are medicines‐related ^9^. Similarly, a previous study in Australia reported that 46% of the hospitalizations among older residents were due to potentially suboptimal use of medicines.^11^ In addition, many aged care residents were on fall‐risk drugs and visited hospital with fractures. Discontinuation or dose reduction in fall‐risk drugs significantly reduces the occurrence of falls in older people.^12^

Since medicines‐related harm frequently occurs in vulnerable older residents, pharmacists working in the aged care sector may play an important role in reducing the incidence of these harms and improving medication use in older people living in RACFs.^3^ In RACFs, the skills of healthcare professionals, particularly when working as a team, can contribute to improved patient outcomes.^13^ A study reported that the lack of accessibility to pharmacists is an important factor affecting medication safety and quality use of medicines in RACFs.^14^ A recent review indicated that pharmacist interventions appear to improve medication safety in older residents based on observed reductions in medicines‐related harm, particularly when the provision of care involves multidisciplinary collaboration.^3^ Many factors can affect the outcomes of evidence‐based interventions; however, the success of implementation efforts depends on a careful assessment of barriers to, and facilitators of, the behavior to be changed.^15^ In Australia, little is known about the barriers and facilitators experienced by pharmacists in reducing medicines‐related harm in RACFs. No study has extensively used a qualitative approach to investigate the views of pharmacists toward reducing the risk of medicines‐related harm in aged care residents. Therefore, we aimed to explore and understand the views of pharmacists providing services in RACFs on medicines‐related harm, harm reduction strategies, associated potential risk factors, and barriers and facilitators to reducing medicines‐related harm in aged care residents.

## 2 METHODS

The qualitative approach selected for this research was informed by grounded theory. We used a qualitative thematic analysis using an inductive approach to explore the views of pharmacists toward reducing the risk of medicines‐related harm in older residents. Face‐to‐face semi‐structured interviews using an interview guide (Table S1) were conducted via Zoom with pharmacists across Australia providing services (e.g., through the provision of Residential Medication Management Reviews (RMMRs), supplying medications to RACFs, or being an embedded/on‐site pharmacist) to RACFs. An RMMR is conducted by an accredited pharmacist and facilitates the quality use of medicines and helps reduce the occurrence of medicines‐related harm in older Australians living in government‐funded RACFs.^16^ The interview guide was prepared based on the literature that aligns with our study objectives. This aided in the development of our list of questions for the pharmacists working in RACFs as well as the identification of significant themes and subjects that were pertinent to our research question. After going through this process, we came up with an outline of the main themes or topics we wanted to address in the interviews. This made it easier to plan and formulate interview questions and ensured that all relevant subjects were covered. We avoided leading questions and questions that can be responded with a simple “yes” or “no” in order to ensure that the questions are clear, succinct, and open‐ended to allow for a deep and in‐depth investigation of the subject. To guarantee that the questions are focused, relevant, and appropriate, we also revised and improved them through expert opinion and feedback from pharmacists providing services to RACFs. Probes, follow‐up enquiries, or other prompts were also included in the questions to nudge respondents to elaborate on their responses and offer more thorough details. In order to find any problems or potential areas for improvement, we also pilot‐tested the interview guide with a small number of pharmacists. The questions covered: thoughts on medicines‐related harm, pharmacist‐led interventions to improving medication safety, harm reduction strategies currently in use, potential risk factors associated with medicines‐related harm, and barriers and facilitators to reducing medicines‐related harm in older residents. Qualitative data were audio‐recorded, transcribed, and analyzed using the thematic analysis approach explained by Braun and Clarke.^17^ All participants had an opportunity to review the text transcription for accuracy within 2 weeks of the interview prior to thematic analysis, although only one participant accepted this offer. The Consolidated Criteria for Reporting Qualitative Studies (COREQ) were followed in the reporting of the research findings.^18^

### 2.1 Participant recruitment

As a guide, it is recommended that qualitative studies require a minimum sample size of at least 12 to be likely to reach data saturation.^19^ A convenience sampling technique was used to recruit 15 pharmacists providing services to RACFs, who gave their consent via an ongoing survey regarding the perceptions and practices of pharmacists toward reducing medicines‐related harms in aged care residents. A web link to the survey questionnaire was shared with the pharmacists via Australian professional pharmacy organizations (e.g., Australian Association of Consultant Pharmacy, Pharmacy Guild of Australia, Professional Pharmacists Australia, and Pharmaceutical Society of Australia), Pharmacy Daily (an online publication), and social media groups (e.g., LinkedIn, Facebook, and Twitter). At the end of the survey questionnaire, pharmacists had an opportunity to complete a separate recruitment form for the follow‐up interview to share their experiences providing services to RACFs. All participants were emailed an information sheet about the research and a consent form prior to the interview. We provided a $50 gift card to each participant.

### 2.2 Data collection

Interviews were conducted between March and April 2022. The following participant demographics were collected: gender, age group, location of participants' residence, employment location, role in aged care, frequency of service to RACFs, experience providing services to RACFs, accreditation status to perform RMMRs, and the number of RMMRs pharmacists performed each month. Zoom interviews were conducted and transcribed by one researcher (SA), who had prior training and experience in conducting interviews for qualitative research. For accuracy, three researchers (MS, GP, and CC) checked interview transcripts. Interview times ranged from 25 to 30 min.

### 2.3 Analysis

The data were analyzed using NVivo QSR 10 after interviews were transcribed. Thematic analysis was performed via inductive coding and the creation of sub‐themes and themes. Data coding was performed independently by two authors (SA and MS). Together, the authors (CC, MS, SA, and GP) discussed and revised the final coding, and sub‐theme and theme wording and structure.

### 2.4 Ethics

Approval for this study was obtained from the Tasmanian Human Research Ethics Committee (Reference: H0026755).

## 3 RESULTS

Fifteen interviews were conducted; 11 pharmacists were providing RMMRs (and one of these also supplied medication to RACFs), one worked as a medication supply pharmacist, two worked as on‐site pharmacists in RACFs, and one worked in a transition care program. Many pharmacists worked in metropolitan areas (*n* = 10, 67%). Most pharmacists were female (*n* = 11, 73%) and were between 30 and 59 years old. Two‐thirds of the pharmacists provided services to more than three RACFs. The median years of experience of providing services to RACFs was 13 years (range, 5–22 years), and 14 pharmacists (93%) were accredited to perform RMMRs. Eight pharmacists (54%) performed more than 20 RMMRs monthly. Pharmacists were from six states of Australia, as detailed in Table 1.

**TABLE 1:** Demographics of participating pharmacists (*N* = 15).

Variable	*n* (%)
Gender	
Male	4 (27)
Female	11 (73)
Age	
20–29	1 (7)
30–39	5 (33)
40–49	2 (13)
50–59	5 (33)
60 and above	2 (13)
Location	
New South Wales	1 (7)
Victoria	7 (46)
South Australia	2 (13)
Tasmania	1 (7)
Western Australia	1 (7)
Queensland	3 (20)
Employment location	
Urban	10 (67)
Rural/Regional	5 (33)
Role in aged care^a^	
Providing RMMRs	11
Supplying medications to RACFs	2
Embedded or on‐site residential care pharmacist	2
Other (Transition Care Program)	1
Providing service to RACFs	
1	1 (7)
2 or 3	4 (27)
More than 3	10 (66)
Experience as a pharmacist providing services to RACFs, median (IQR)	13 (5–22)
Accredited to conduct RMMRs	
Yes	14 (93)
No	1 (7)
Number of RMMRs performing each month	
< 10	2 (13)
10–20	3 (20)
> 20	8 (54)
Not applicable (do not conduct RMMRs)	2 (13)

Abbreviations: IQR, interquartile range; RACF, residential aged care facility; RMMR, Residential Medication Management Review.

^a^ Did not calculate the percentage as some pharmacists provided more than one service.

Several themes were identified regarding medicines‐related harm in aged care residents (Table S2).

### 3.1 Common medicines‐related harms

Most pharmacists reported that falls, anticholinergic effects, and adverse effects on the central nervous system (CNS), such as sedation and confusion, were common medicines‐related harms in older residents. Many pharmacists discussed that the occurrence of fall was a serious medicines‐related injury in frail older residents with multiple comorbidities.

*“…when falls in aged care occur, that's the one that can cause serious harm to patients and can have lasting effects.” (P1, RMMR pharmacist)*.

Pharmacists expressed that general practitioners (GPs) prescribe anticholinergic‐type medicines, but often overlook the impact of anticholinergic effects.

*Ah, probably the anticholinergic effects, because a lot of GPs are not looking for that. When you're looking specifically at, say, anticholinergic drug burden. You know, there's a lot of evidence out there for reducing any cholinergic side effect drugs. (P12, RMMR pharmacist)*.

Other pharmacists reported that medicines acting on the nervous system were causes of sedation and confusion, with the subsequent risk of falls and their sequelae.

*Probably drug‐induced sedation, yeah, you know, increasing the risk of confusion increasing the risk of fall. (P14, RMMR pharmacist)*.

Several pharmacists pointed out that strong anticholinergics were involved in causing severe confusion as these are CNS‐acting medicines.

*Probably the use of anticholinergic agents such as oxybutynin, where they get very, very confused with it. (P4, RMMR pharmacist)*.

### 3.2 Risk factors for harm

Medicines‐specific risk factors for harm included polypharmacy, potentially inappropriate medications (PIMs), anticholinergic effects of drugs, sedative load, lack of reconciliation of medicines, and the risk of commencing new drugs. Patient‐specific risk factors included renal impairment, frailty, and a lack of aged care staff engagement with residents and families.

Many pharmacists described the use of multiple medicines as a common significant risk in aged care residents. Similarly, pharmacists pointed out that sedatives and antipsychotics are overprescribed and not frequently reviewed, thus increasing the risk of medicines‐related harm.

*“…there are huge rates of polypharmacy, you know, sedative medications and probably things like antipsychotic, we're overusing it and they're not getting reviewed often enough.” (P1, RMMR pharmacist)*.

Some pharmacists believed that the lack of aged care staff engagement with the residents and families is an important risk factor for harm.

*“.. the biggest risk is that people are not engaging with the individual resident and their families. I think it's the staff, I think it's not engaging with the individual. I mean the aged care staff, not qualified nurses, and care workers giving out medicines.” (P10, RMMR pharmacist)*.

Pharmacists expressed that the inappropriate prescribing of medicines increases the risk of harm in older residents.

*We want the best medication regimen for a particular person. […] multiple inappropriate medications is probably the most important factor that's causing and resulting in adverse outcomes in our aged care sector. (P8, RMMR pharmacist)*.

Pharmacists said that the reconciliation of medicines is crucial as medicines are not properly reviewed after residents' admission into RACF. Additionally, sometimes aged care residents take medicines for several months or years that were earlier prescribed for a short duration and it occurs because of infrequent review of medicines.

*Risk factors for medicines‐related harm like accurate reconciliation of medications on admission because of discrepancies in chart is important because people get started on medications leading up to admission into aged care. Typically, they don't get stopped appropriately or reviewed appropriately. Sometimes a short course of medication is prescribed, but then it persists unquestioned for months or years. And that is suppose, again, that's monitoring, but also has a degree of reconciliation admission, making sure there's an accurate medication list. (P13, RMMR and supply pharmacist)*.

Pharmacists expressed that the initiation of new medicines increases the incidence of falls in frail older residents.

*Most common risk factor, I think, commencing new drugs. So, you know, they'll commence new drugs. They're frail, they're physically frail. So, they're physically frail in their mobility. And they're also frail in their ability to, you know, they're more at risk of acute renal failure. There are more risk to adverse effects from the drugs. And the consequence of that is that they might have a fall. (P14, RMMR pharmacist)*.

Some pharmacists reported that older residents with renal impairment are more prone to medicines‐related harm and implied medication dosing was not adequately reviewed.

*I think renal function is a big risk, medicines often continued too higher doses for their renal function. (P10, RMMR pharmacist)*.

Pharmacists also discussed that frailty in older people increases the incidence of falls and other health‐related complications.

*Frailty. Because if they're frail and old, and I suppose I think in aged care and thinking falls again, I've got to look at it a more broader point. In older patients, things can go wrong really quickly. And you know, if something happens they become confused, something like that there's all those co‐morbidities and for older, frail patients, they seem to happen more frequently than for other patients. (P11, Pharmacist involved in transition care program)*.

### 3.3 Barriers to improving medication safety

#### 3.3.1 Aged care facility barriers

Pharmacists providing services to RACFs experienced various barriers, such as aged care staff not accepting pharmacists' recommendations, COVID‐19 restrictions, family influence in managing residents' medications, and aged care staff burnout.

Pharmacists discussed their concerns regarding the lack of support from aged care staff due to lack of awareness about medicines and the deprescribing process.

*Not understanding what we're trying to do. I think, they think we're trying to stop medicines, you know. I think they understand sometimes that the medicines are not appropriate. (P11, Pharmacist involved in transition care program)*.

Pharmacists said nurses in RACFs may manage medications of older residents via an antimicrobial stewardship program, but they lack the fundamental knowledge required to implement this program.

*My biggest issue is, I suppose nursing staff have been given control of basically medication stewardship programs in residential aged care. And they don't have the clinical understanding or the knowledge to apply stewardship programs in medication management for residents in aged care. (P13, RMMR and supply pharmacist)*.

Some pharmacists experienced COVID‐related restrictions in RACFs that impeded access to information required for conducting medication reviews.

*It's been COVID, because we've been having to work remotely. And the other thing that is basically the lack of the computerised medication charts, that the document we're relying on are mostly faxed prescriptions, not all of my homes have allowed us electronic access. So, we're falling behind with that. (P4, RMMR pharmacist)*.

Pharmacists also reported that many GPs and aged care staff resigned from their job during COVID‐19 as they faced a burnout crisis due to overburden and inadequate support.

*We've heard a huge number of people resigning. From physicians that have been overworked, they've had insufficient support over COVID times. And they just can't let they really had burnout. So, we've had huge numbers of staff leaving on top of guilt. (P4, RMMR pharmacist)*.

Another pharmacist discussed the influence of residents' family members on decisions made at case conferences regarding deprescribing medicines.

*It's the family. You know, I've been in a couple of case conferences where we've reduced medications, because somebody is increasing frailty, but the family then come along and go, nope, I want Mum to back on all that medication again. (P12, RMMR pharmacist)*.

#### 3.3.2 GPs and system barriers

GPs and systems‐related barriers included lack of funding for pharmacists in aged care and insufficient remuneration for pharmacists, GPs not accepting pharmacists' recommendations, infrequent medication reviews/poorly written RMMRs by pharmacists, absence of regular aged care staff and GPs, and supply pharmacists being unable to identify drug‐related problems. All pharmacists in this study believed that lack of funding or insufficient remuneration for pharmacists was the most common system‐related barrier to improving medication safety in aged care residents.

*For accredited pharmacists, and probably pharmacy owners and things, maybe the funding isn't quite there […] because there's not enough funding and the pharmacies are busy that most of the time, you know, you can be in the facility for a little bit, but then you need to go back to the pharmacy to do usual work in the community pharmacy. (P1, RMMR pharmacist)*.

*Rule states 2 years, but I've been trying to do them earlier, every two years is just too far apart. For me, the business rules were just a joke when they changed it from one year to two years. The only reason they changed it was because funding was running out. (P2, RMMR pharmacist)*.

Some pharmacists reported that most of their recommendations are not actioned by GPs.

*Barriers are when doctors just don't want to make any changes. Doctors not taking my advice on board, that would be the biggest barrier. Some doctors are just using the program as an income and not make any changes and I get really, really frustrated with that. (P2, RMMR pharmacist)*.

Pharmacists also mentioned that infrequent and poorly written medication reviews impede efforts to improve medication safety in older residents.

*RMMR is a great resource of information. Some people don't write very good RMMRs, so GPs see those and stop taking any notice of what pharmacists are saying. So, if there was some standardisation, perhaps and quality indicators, or some sort of gathering of data from RMMRs, I think that would help us all strive toward better report writing so that the GPs could rely on that information. So that deprescribing could happen. (P5, (P13, RMMR pharmacist)*.

### 3.4 Facilitators to improving medication safety

#### 3.4.1 Aged care facility facilitators

Three facility‐level facilitators emerged from the analysis: collaboration and strong relationships with aged care staff, residents, and residents' family; provision of education and awareness to the aged care staff, residents, and residents' family; and the Medication Advisory Committee meetings within RACFs.

Collaboration and strong relationships with aged care staff, residents, and residents' family emerged as key facilitators to improving medication safety in older residents. Pharmacists believed that they could effectively deprescribe medicines and reduce harm via collaborative efforts with other stakeholders involved in caring for residents.

*I don't think it's necessarily just going to be pharmacists that are going to be able to solve the problem, I think it's going to come through a better collaborative approach between the nursing staff and carers and pharmacists, as well as other allied health professionals. I think it's going to be a model where there's increased access and collaboration between lots of different healthcare professionals in aged care. (P1, RMMR pharmacist)*.

Some pharmacists discussed that the provision of education is crucial for the aged care staff, but they do not frequently receive education such as the provision of guidance about the irrational use of antibiotics.

*Education for staff is one that is just so essential. I don't think it happens very often. Even things like providing advice about when to use antibiotics, things like that. (P11, Pharmacist involved in transition care program)*.

Pharmacists also reported Medication Advisory Committee meetings (a group of advisors to RACFs) as one of the facilitators to improving medication safety in RACFs.

*Communication is sent out to the prescribers, you know, everything gets agreed on in the Medication Advisory Committee, it's quite good. So, at those meetings, for example, have polypharmacy reported to the prescribers and we give them like the national average. And then we give them where we're at with which facility, they get the data. And it's actually been highly successful, the doctors have been totally on board with deprescribing. And I think that has been a significant help along with RMMRs. (P14, RMMR pharmacist)*.

#### 3.4.2 GPs and systems facilitators

Pharmacists reported the following GPs and systems‐related facilitators to improve medication safety: collaboration with GPs, provision of education to the GP, provision of funding/increased remuneration for pharmacists, medication chart review or RMMRs, pharmacists with adequate clinical therapeutics experience, and training for pharmacists. Provision of funding or increased remuneration for pharmacists emerged as a key system‐related facilitator to improving medication safety in older residents. Pharmacists reported that the provision of sufficient funds for conducting medication reviews and extra compensation will engage pharmacists more in improving medication safety.

*Increase funding definitely, say extra remuneration would be a big factor. I think increased funding would help because you'd be able to spend more time and more follow up, which I think would lead to a reduction in those harms. (P1, RMMR pharmacist)*.

Some pharmacists reported that collaboration with GPs is crucial in improving medication safety.

*We need to improve our working relationships with the GPs and with the staff in the clinics, so that they're more aware of what we're trying to do. (P11, Pharmacist involved in transition care program)*.

Pharmacists also mentioned that the provision of education to GPs is critical as they are not experts in pharmacotherapy.

*Education of GPs as they don't have the medication, and the pharmacological insights that we probably do as pharmacists, they can still be looking out for particular side effects. (P12, RMMR pharmacist)*.

Many pharmacists believed that the pharmacist‐led medication review is effective in identifying and reducing medicines‐related injuries among both existing and newly admitted aged care residents.

*I think like RMMRs are obviously a big thing that's going to help medication safety. I think just by reviewing medications and things you can reduce, you know, inappropriate medication use and then reduce, you know, medication‐related harm from that. (P1, RMMR pharmacist)*.

Pharmacists also highlighted the importance of clinically trained pharmacists in RACFs. Additionally, mentoring by an experienced pharmacist and residency training in a tertiary care setting are crucial for pharmacists that help to acquire the required clinical experience, thus improving patient‐centered care.

*The most important thing is having the presence of a pharmacist, but not just any pharmacist, I think it has to be a very experienced pharmacist, who is not a pharmacist that just cut and pastes out of literature and actually have mentoring. And from very experienced clinical pharmacists, I really think how pharmacists that work in aged care, that come in and out or like are unlikely to have much of an impact, I think, a pharmacist that is there and present and is current up to date and experienced and has a residency program, preferably in a university associated teaching hospital where they can gather a lot of clinical applied therapeutics experience, because it's the applied therapeutics that is missing in our profession. We actually are very good at theory, but not so good at applying it for a person‐centred approach. (P8, RMMR pharmacist)*.

A few pharmacists believed that aged care pharmacists should acquire further specialized training with an emphasis on aged care.

*Some sort of increased training, maybe I'm not sure if that will be during the undergraduate degree. If there's a little bit more focused on aged care, or I guess as accredited pharmacists, you do additional training, which is great […] If you're supplying medications to an aged care facility, you should maybe if there was like a training course for those pharmacists, which wouldn't have to be much but you say that these are the harm that are things in aged care, and this is what we should be looking at would be good as well. (P1, RMMR pharmacist)*.

### 3.5 Resources for reducing medicines‐related harm

Participants discussed numerous resources for reducing medicines‐related harm in older residents, such as Beers criteria, clinical resources (e.g., books and guidelines), drug burden index calculator, the screening tool of older people's prescriptions and screening tool to alert to right treatment (STOPP/START), drug interaction checkers, pain medicines opioid calculators, and the CHA₂DS₂‐VASc calculator for patients with atrial fibrillation. Many pharmacists pointed out that they mostly utilized Beers criteria to avoid inappropriate prescribing and medicines‐related harm.

## 4 DISCUSSION

Pharmacists can play a key role in reducing the occurrence of medicines‐related harm in older people living in RACFs.^3^ Their insights are needed before developing an effective tailored intervention toward reducing the risk of medicines‐related harm in this cohort.

Pharmacists reported that polypharmacy, PIMs, anticholinergic effects of drugs, and sedative load are medicines‐related risk factors for harm in older residents, as similarly identified in previous reports.^20^, ^21^, ^22^ In this study, pharmacists also reported other medicines‐related risk factors such as medicines reconciliation and the risk of commencing new drugs. Medication reconciliation is recognized as an effective approach for preventing medicines‐related harm.^23^ Medication order discrepancies can happen during the transition of care due to infrequent communication between nurses, pharmacists, and GPs.^24^

Similarly, effective communication between pharmacists, GPs, and aged care staff based on evidence‐based information could increase the uptake of pharmacists' recommendations, such as discontinuation of unnecessary medicines and dose reductions.^25^ Pharmacists often stated that GPs and aged care staff do not accept their recommendations, impeding efforts to improve medication safety in older residents. Pharmacists stated the need for strong interdisciplinary collaboration between GPs, pharmacists, and aged care staff, to improve medication safety in RACFs. A lack of communication between healthcare professionals caring for older residents is a significant factor influencing medication safety and the quality use of medicines within RACFs.^14^ A collaborative pharmacist‐initiated medication review with a GP exhibited a significant reduction in medicines‐related injury in aged care residents.^26^ A multidisciplinary case conference between GPs, pharmacists, and aged care staff is an effective strategy for improving medication safety among older residents.^27^ The provision of training in effective communication between healthcare professionals providing services to RACFs may improve the quality use of medicines in older residents.

Overall, pharmacists believed that medicines‐related injury is multifactorial and a significant problem in Australian RACFs. Pharmacists in our study frequently encountered falls in frail older residents with multiple comorbidities. Furthermore, pharmacists discussed that inappropriate use of antipsychotics and anticholinergics increased the risk of medicines‐related harm in older residents. A previous study in Finland reported that multiple comorbidities are associated with the risk of recurrent falling.^28^ Fall‐risk drugs, such as anticholinergics, opioids, benzodiazepines, antidepressants, and antipsychotics, are frequently prescribed for older residents.^29^ It is crucial to minimize use of these drugs to prevent falls and fall‐associated injuries.^12^ A recently published Australian Aged Care Royal Commission report recommended stricter requirements for prescribing antipsychotics for people living in RACFs.^30^ The Royal Commission advocated that older residents should be individually reviewed by a psychiatrist preceding the initiation of antipsychotics.^30^ The successful RedUSe intervention, comprising multidisciplinary case review, staff education, and audit and feedback, can be implemented in Australian RACFs to reduce the irrational prescribing of antipsychotics.^31^

Another facilitator to reducing medicines‐related injury was the provision of relevant education to all stakeholders involved in managing older residents. The Royal Commission highlighted that Australia's aged care staff frequently lack the required skills and training to cater for the needs of aged care residents.^32^ Furthermore, inadequate skill mix and training are primary causes of substandard care in the aged care sector.^32^ Evidence indicates that the provision of education to aged care staff improves medication safety in older residents.^3^ A previous study in Australia reported that teaching sessions for at least 1 h improved the aged care staff's knowledge of medication management.^33^

In our study, pharmacists cited the importance of frequent medication reviews for identifying and reducing medicines‐related harm among both existing and new older residents. Likewise, pharmacists reported that infrequent and poorly written medication reviews are barriers to improving medication safety in aged care residents. The Royal Commission recommends conducting medication reviews immediately after the admission of older patients in RACF and every 12 months thereafter.^32^ Furthermore, medication reviews need to be conducted more frequently if there has been a change to the residents' health condition or medication regimen.^32^ A well‐written medication review minimizes medicines‐related issues and optimizes patients' therapeutic outcomes.^34^ Furthermore, pharmacists providing services in aged care should be equipped with the clinical therapeutic experience required for conducting and writing a comprehensive medication review.^35^

The systematic problems such as inadequate funding for the aged care sector contribute to improper care of older residents.^30^ Pharmacists indicated that insufficient funding and remuneration for pharmacists are barriers to improving medication safety in older residents. The Royal Commission also acknowledged insufficient government funding for aged care and indicated that the provision of care for older residents is influenced by insufficient funding arrangements.^30^ Pharmacists are pharmacotherapy experts and are well‐suited to conduct medication reviews and ascertain inherent causes of medicines‐related problems and provide solutions to prevent them.^36^ Therefore, the provision of funding for pharmacists providing services to RACFs and education to aged care staff are critical in improving medication safety in aged care residents. The Australian government announced a $345.7 million investment for on‐site pharmacists in RACFs on March 25, 2022, with the goal of improving medication safety and quality of care for older residents.^37^

### 4.1 Strengths and limitations

This study is the first of its kind that explored the views of Australian pharmacists providing services in RACFs toward reducing the risk of medicines‐related harm in aged care residents. This study also has certain limitations. A qualitative study may not demonstrate whether pharmacists can improve health outcomes in older residents, and a randomized controlled trial is needed to yield positive results. Other healthcare professionals were not interviewed; only pharmacists were, which may have affected the extraction of other barriers and facilitators that may have existed but were missed. However, the interpretation of the interviews could be subjective due to the qualitative nature of the study; therefore, some caution is needed.

### 4.2 Implications for clinical practice and future research

Since medication use is a serious ongoing concern in Australian RACFs, this qualitative study facilitated exploring safety issues and recommendations for improvement. The study findings imply that adequate funding, provision of customized training to pharmacists, and their mentoring through experienced clinical pharmacists may improve medication safety in RACFs. Medicines use in older residents can be optimized via collaboration between GPs, pharmacists, and aged care staff, thus improving residents' health outcomes with multiple comorbidities. Effective communication between GPs, pharmacists, and aged care residents can help ensure that the residents receive the best possible care. Electronic health records, medication management reviews, telehealth, collaborative care models, clear communication policies, and medication charts are a few examples of tools, resources, or policy changes that may improve communication in this situation. To support healthcare professionals (including GPs, nurses, and pharmacists) through the process of optimizing medicine use in RACFs, there is a paucity of evidence‐based training packages in practice. Therefore, developing such a training package would be a valuable future direction for researchers in this area. Future research is also needed to ascertain the impact of educating GPs and aged care staff about medication safety on the uptake of pharmacists' recommendations, as well as the reasons for not accepting recommendations.

## 5 CONCLUSIONS

Pharmacists believed that the irrational use of medicines increases harm in aged care residents and medicine‐specific (e.g., sedative load) and patient‐specific risk factors (e.g., renal impairment) are associated with injuries in residents. To reduce medicines‐related harm, the participants highlighted the need for increased funding for pharmacists, improving all stakeholders' awareness about medicines‐associated harms through education, and ensuring collaboration between healthcare professionals caring for aged care residents.

## FUNDING INFORMATION

This research received no specific grant from any funding agency in public, commercial or not‐for‐profit sectors.

## CONFLICT OF INTEREST STATEMENT

The authors declare that they have no conflict of interest.

## Supporting information


Data S1.

## Data Availability

All data are available in tables and Data S1.
