# Supplementary material for: Exploring Australian pharmacists' views toward reducing the risk of medicines‐related harm in aged care residents
Source: Pharmacol Res Perspect. 2023 May 24;11(3):e01104. doi: 10.1002/prp2.1104 (PMC10207935; doi:10.1002/prp2.1104)
Supplement: Supplementary file 1 — Data S1. [file PRP2-11-e01104-s001.docx]

**Table S1** Interview guide

Hello, my name is Sheraz Ali, and I am a PhD candidate at the University of Tasmania. Thank you for agreeing to participate in this interview.

I am going to ask you several questions today. If any questions are not clear, please let me know. All information collected will be strictly confidential. I would like to record our chat today so that we have an accurate record of it, and I don’t have to rely on my memory. Are you happy for the interview to be recorded? Any questions before we start the chat?

| **Topic** | **Questions** | **Prompts/probes** |
| --- | --- | --- |
| General thoughts | Can I start by asking you what your thoughts are about medicines-related harms in the aged care setting? | What is the most common medicines-related harm in the aged care setting that you usually encounter during your practice? and how can we prevent this harm? |
| Information about risk factors | What are your thoughts about the risk factors for medicines-related harms? | What is the most common important risk factor for medicines-related harms? and why?  Based on your experience, is there any other important risk factor for medicines-related harms? If yes  Why it is important? |
| Resources | What helps you to reduce medicines-related harm in aged care residents? | Are there any resources or tools that help you? Do you use these tools much? Why do you use these tools?  Other pharmacists, aged care facility staff, community pharmacists, doctors – in what way do they help, and in what ways are they barriers? |
| Barriers | What barriers prevent you from reducing medicines-related harms in aged care residents? | What is the biggest thing standing in your way? Your biggest frustration/problem/worry?  What issues do you have with aged care facility staff, community pharmacists, doctors, patients, and patients’ guardians?  Do pharmacists have time to review residents’ medication profiles?  How often do they get to see residents’ medication profiles?  Do they get time to discuss with doctors or other healthcare professionals in the RACFs? |
| Pharmacist-led interventions | What are the pharmacists’ interventions that can improve medication safety in RACFs? | Do you think aged care patients, nurses, and doctors will benefit or be irritated by pharmacist-led interventions? Neither?  Do you think patient outcomes may be affected due to pharmacist-led interventions? how so? |
| Facilitators | What factors can facilitate involving pharmacists more in improving medication safety in RACFs? | Does it have any impact on reducing the incidence of medicines-related harms or improving medication safety in older residents?  is there any other facilitator? If yes  Which one? |
|  | What opportunities are open to pharmacists to find out if their services are providing benefit to aged care residents? | Do pharmacists have an opportunity to improve their services based on feedback from patient improvement or not, or from other aged care staff?  is remuneration for their services a problem?    Do people respect and act on their reports and suggestions? |

That’s all the questions I have for you- Is there anything else that you feel important, and we have not discussed today?

**Table S2** Themes and subthemes.

|  |
| --- |
| **Barriers to implementing medication safety** |
| Aged care facility-related barriers |
| Aged care staff burnout |
| Aged care staff do not accept pharmacists' recommendations |
| COVID-19 restrictions |
| Family influence in managing medications of residents |
| GPs and systems-related barriers |
| Absence of regular aged care staff and GP |
| GPs do not accept pharmacists' recommendations |
| Infrequent medication reviews / Poorly written RMMRs by pharmacists |
| Lack of funding for pharmacists in aged care or insufficient remuneration for pharmacists |
| Supply pharmacists are unable to identify drug-related problems |
| **Facilitators to improving medication safety** |
| Aged care facility-related facilitators |
| Collaboration and strong relationships with aged care staff, residents, and family |
| MAC meetings |
| Provision of education and awareness to the aged care staff, residents, and residents' family |
| GPs and systems-related facilitators |
| Collaboration with the GPs |
| Provision of education to the GPs |
| Provision of funding / Increased remuneration for pharmacists |
| Pharmacists with clinical therapeutics experience |
| Training for pharmacists |
| Medication chart review or RMMRs |
| **Most common medicines-related harms** |
| Adverse effects on nervous system (e.g., Sedation and confusion) |
| Anticholinergic effects |
| Dizziness |
| Falls |
| Weight loss |
| **Patient and medicines' specific risk factors for harm** |
| Renal impairment |
| Sedative load |
| Frailty |
| Lack of aged care staff engagement with the residents and their families |
| Anticholinergic effects of drugs |
| Inappropriate reconciliation of medicines |
| Polypharmacy |
| Potentially inappropriate medications |
| Risk of commencing new drugs |
| **Potential medicines involved in harms** |
| Anticholinergics |
| Antipsychotics |
| Diuretics |
| Sedatives |
| **Resources to reducing medicines-related harms** |
| Beers criteria |
| CHA₂DS₂-VASc Calculator |
| Clinical resources (Books, guidelines) |
| Drug burden index calculator |
| Drug interaction checker |
| Pain Medicines Opioid calculator |
| STOPP/START tool |

COVID, coronavirus disease; GP, general practitioner; RMMR, residential medication management review; STOPP/START, screening tool of older people's prescriptions and screening tool to alert to right treatment; MAC, Medication Advisory Committee
